# What is an evidence map? A systematic review of published evidence maps and their definitions, methods, and products

**DOI:** 10.1186/s13643-016-0204-x

**Published:** 2016-02-10

**Authors:** Isomi M. Miake-Lye, Susanne Hempel, Roberta Shanman, Paul G. Shekelle

**Affiliations:** Evidence-based Synthesis Program (ESP) Center, Veterans Affairs Greater Los Angeles Healthcare System, 11301 Wilshire Blvd, Los Angeles, CA 90073 USA; Department of Health Policy and Management, Fielding School of Public Health, University of California, Los Angeles, 640 Charles E Young Dr S, Los Angeles, CA USA; Southern California Evidence-based Practice Center, RAND Corporation, 1776 Main St, Santa Monica, CA 90401 USA; Department of Medicine, David Geffen School of Medicine, University of California, Los Angeles, 10833 Le Conte Ave, Los Angeles, CA 90095 USA

**Keywords:** Evidence map, Evidence synthesis, Systematic review

## Abstract

**Background:**

The need for systematic methods for reviewing evidence is continuously increasing. Evidence mapping is one emerging method. There are no authoritative recommendations for what constitutes an evidence map or what methods should be used, and anecdotal evidence suggests heterogeneity in both. Our objectives are to identify published evidence maps and to compare and contrast the presented definitions of evidence mapping, the domains used to classify data in evidence maps, and the form the evidence map takes.

**Methods:**

We conducted a systematic review of publications that presented results with a process termed “evidence mapping” or included a figure called an “evidence map.” We identified publications from searches of ten databases through 8/21/2015, reference mining, and consulting topic experts. We abstracted the research question, the unit of analysis, the search methods and search period covered, and the country of origin. Data were narratively synthesized.

**Results:**

Thirty-nine publications met inclusion criteria. Published evidence maps varied in their definition and the form of the evidence map. Of the 31 definitions provided, 67 % described the purpose as identification of gaps and 58 % referenced a stakeholder engagement process or user-friendly product. All evidence maps explicitly used a systematic approach to evidence synthesis. Twenty-six publications referred to a figure or table explicitly called an “evidence map,” eight referred to an online database as the evidence map, and five stated they used a mapping methodology but did not present a visual depiction of the evidence.

**Conclusions:**

The principal conclusion of our evaluation of studies that call themselves “evidence maps” is that the implied definition of what constitutes an evidence map is a systematic search of a broad field to identify gaps in knowledge and/or future research needs that presents results in a user-friendly format, often a visual figure or graph, or a searchable database. Foundational work is needed to better standardize the methods and products of an evidence map so that researchers and policymakers will know what to expect of this new type of evidence review.

**Systematic review registration:**

Although an a priori protocol was developed, no registration was completed; this review did not fit the PROSPERO format.

**Electronic supplementary material:**

The online version of this article (doi:10.1186/s13643-016-0204-x) contains supplementary material, which is available to authorized users.

## Background

There is growing variation in evidence synthesis methodology to meet the different objectives evidence synthesis can support. The classic systematic review and meta-analysis are both rigorous and produce detailed information about narrow questions, but they are resource intense and the work burden limits the scope of what can be covered [1]. To meet a variety of user needs, offshoots of the classic model have been developed within the evidence synthesis realm; for example, rapid reviews cater to more urgent deadlines but may not adhere to all the methods of a systematic review [2], scoping reviews accommodate larger bodies of literature for which detailed synthesis is not needed [3], and realist reviews specialize in exploring how complex interventions work and frequently include evidence excluded from classic systematic reviews [4].

These new variants on the classical systematic review are at various phases in development. Determining the unique contributions and methods of each of these new synthesis method offshoots is a challenge. Systematic reviews and meta-analyses have a standardized process for conduct and reporting, codified in the Institute of Medicine standards and Preferred Reporting Items for Systematic Reviews and Meta-Analyses (PRISMA) reporting guidelines [5, 6]. The Realist And Meta-narrative Evidence Syntheses: Evolving Standards (RAMESES) publication standards for realist syntheses and meta-narrative reviews were published in 2013 [7, 8], and scoping review reporting guidance is underway as of 2014 using the Enhancing the QUALity and Transparency Of health Research (EQUATOR) Network [3]. Rapid reviews, also sometimes referred to as evidence summaries [9], have received increased interest, with a journal series and dedicated summit in 2015 [10, 11], as well as multiple articles on rapid review methodology [9, 12, 13], but no official standards have been released.

Evidence mapping is the newest of these new evidence review products. In 2002, there were no published evidence maps, and as recently as 2010, only ten such publications could be identified. In addition, evidence mapping has yet to undergo the scrutiny and development of these other methodologies, and it is not clear if those authors who are using the term are using a methodology unique from other developing methods. Both evidence maps and scoping reviews set out to map the literature. A scoping review recently was described as “a form of knowledge synthesis that addresses an exploratory research question aimed at mapping key concepts, types of evidence, and gaps in research related to a defined area or field by systematically searching, selecting, and synthesizing existing knowledge” [3]. They recommend a process described by Arksey and colleagues and enhanced by Levac and colleagues; these steps were also referenced in other publications comparing methods of evidence synthesis as the standard for scoping reviews [14–16]. Thus, there is some consensus surrounding the scoping review method and its components. A 2013 publication attempting to distinguish between the two methodologies concluded that scoping reviews include “a descriptive narrative summary of the results” whereas evidence maps identify evidence gaps, and both use a tabular format to depict a summary of literature characteristics [14]. Furthermore, multiple publications lay out differing recommendations for evidence maps, complicating this effort [14, 17, 18]. Hence, earlier attempts by Schmucker [14] and Snilstveit [15] to characterize evidence map methods and products relied on small numbers of evidence map publications in determining their results and reached conflicting conclusions. Schmucker and colleagues' review included seven evidence maps [14]; Snilstveit discussed three examples while primarily focusing on distinguishing these from what they termed an evidence gap map [15]. Since then, more evidence maps have been published, increasing the ability to determine what constitutes an “evidence map,” either in terms of methods or products. In 2014 alone, eleven evidence maps were published.

Our objectives are to identify and systematically review published evidence maps and to assess commonality and heterogeneity and to determine whether additional work is warranted to try to standardize methods and reporting. We compare and contrast the presented definitions of evidence mapping, the domains used to classify data in evidence maps, and the form of the evidence map takes.

## Methods

### Literature search

The librarian on our team, RS, conducted an electronic search of ten databases (PubMed, PsycINFO, Embase, CINAHL, Cochrane Database of Systematic Reviews (CDSR), Cochrane Central Register of Controlled Trials (CENTRAL), Cochrane Database of Abstracts of Reviews of Effects (DARE), Cochrane Methodology Register (CMR), SCOPUS, and Web of Science) from inception to 8/21/2015 for publications relating to our objective by using the search terms "evidence map" OR "evidence mapping" OR "evidence maps" OR "mapping evidence" OR “evidence map*” in a search of titles and abstracts. Because evidence mapping is a relatively new method in the biomedical literature synthesis repertoire, no Medical Subject Headings (MeSH) term is available. There were no language restrictions or restrictions on study design. Additional studies were identified through reference mining of identified studies and expert recommendations.

### Study selection

Two reviewers independently screened titles and abstracts for relevance and obtained full text articles of publications deemed potentially relevant by at least one reviewer. Full text articles were screened against predetermined inclusion criteria by two independent reviewers, and any disagreements were reconciled through team discussion. To be included, authors must have presented results (i.e., no protocols were included) with a process called evidence mapping or figure called an evidence map. Because of the methodological focus of this review, any patient population, intervention, comparator, outcome, and setting were included.

### Data abstraction

For all included publications, the following data were abstracted: use and definition of the term “evidence map,” research question or aim, search methods and years, number of citations included in the evidence map, and country of origin. The unit of analysis was also abstracted, since some maps considered all literature citations for inclusion, whereas others included systematic reviews only or aggregated all publications originating from the same study into one unit. For publications presenting maps, the domains used to classify studies in the map were also abstracted (e.g., interventions, outcomes, and literature size). Data were abstracted by one reviewer using a standardized form and verified by the second reviewer. The form was piloted and refined by both reviewers prior to abstraction. We applied no quality assessment criteria since we are unaware of any that exist for evidence map methods.

### Data synthesis

Data were narratively synthesized in three sections, discussing key characteristics of definitions presented, domains used to classify literature in the mapping process (e.g., interventions), and what form an evidence map or evidence mapping methodology takes. Within this latter section, publications were grouped by whether they (1) presented a figure or table explicitly called an “evidence map” or called the results evidence mapping in the publication itself, (2) referred to an online database as the evidence map, or (3) said they used a mapping methodology but did not present a figure or table. No statistical analysis was planned or executed, given the focus on reporting of methods rather than a specific outcome and the heterogeneous nature of the included health topics. We created tables to summarize data on included articles to support the narrative synthesis.

## Results

**Fig. 1:**
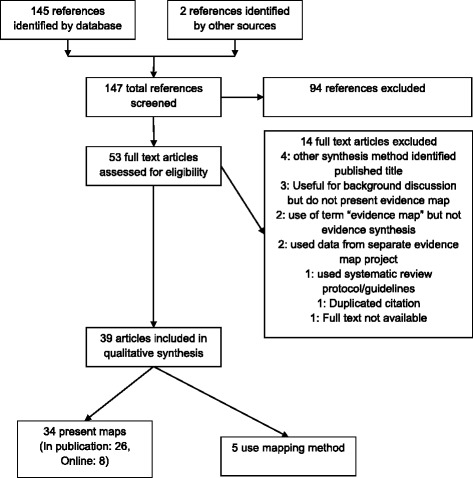
Literature flow

The total search results identified 145 titles through 8/21/2015. Reference mining and expert recommendations yielded one title each for a total of 147 titles for screening. Of these titles, we included 53 potentially relevant publications after title and abstract screening. Fourteen of these publications were rejected after full text review. Four of the excluded publications identified themselves as another type of synthesis (i.e., scoping review, realist synthesis, or systematic review) in their titles [19–22]. Three excluded publications presented discussions of evidence mapping and have been incorporated into discussion where relevant [14, 15, 23]. Two publications used the term “evidence map” outside the evidence synthesis methodology context (e.g., in the context of reporting the results of a hazard assessment) [24, 25]. Another two publications used evidence from an evidence map that was created in a separate project [26, 27], one full text publication was not available [28], one publication was a duplicate citation with another previously screened citation [29], and the final study excluded explicitly stated that they used a standard systematic review protocol developed according to systematic review guidelines [30]. Having met inclusion criteria, thirty-nine publications were included at the full text review. Of these, 34 publications presented evidence maps explicitly and five publications used a mapping methodology without presenting a map [18, 31–34] (see Fig. 1 for literature flow and Additional files 1 and 2 for PRISMA checklist and flow). The publications with explicit maps included those that presented the map in the publication (*N* = 26) [35–60], and those that discussed a map only available online (*N* = 8) [16, 17, 61–66].

**Fig. 2:**
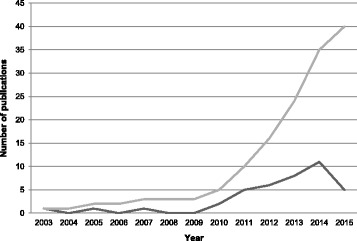
Number of publications per year. The *dark gray line* denotes the number of publications in any given year, and the *light gray line* denotes the cumulative number of publications over time

Included publications came from the USA (*N* = 19), Australia (*N* = 10), the UK (*N* = 7), Canada (*N* = 2), and Japan (*N* = 1). Most publications are very recent, with no publications before 2003 (see Fig. 2). Since the last systematic review of evidence maps, conducted by Schmucker and colleagues during 2013, an additional 24 evidence maps have been published, doubling the literature quantity. More details on data extracted from included publications can be found in Table 1.

**Table 1:** Table of included studies

Author, year	Research question or aim	Unit of analysis	Search methods, years	Country of origin^a^	Citations for definition
Explicit, published (*n* = 26)					
Althuis, 2013 [35]	We intend to identify and summarize the study designs, populations, and outcome measures involved in sugar-sweetened beverage research, providing a foundation for (1) a better understanding of the existing mix of studies, (2) identifying research gaps informing future studies on SSB, and (3) focused research synthesis questions appropriate for systematic review and meta-analyses.	Original study (intervention or cohort studies, *n* = 59)	Followed PRISMA guidelines, multiple databases with standard search terms, reference mined; 1966–2012	USA	Bragge 2011, Levac 2010, Arksey 2005, Katz 2003, Gough 2012
Antsee, 2011 [40]	To identify gaps among the many contemporary studies on HIV prevention from countries with possible relation to HIV in the UK.	Publications (*n* = 716 systematic reviews, RCTs, other primary research articles)	Standard search in multiple databases; 2006–2009	UK	Shepherd 2007, GEM
Bailey, 2014 [44]	To investigate and quantify the nature and distribution of existing high-quality research on the prevention and treatment of eating disorders in young people using evidence mapping methodology.	Trials (*n* = 197), systematic reviews (*n* = 22), follow-up studies (*n* = 10)	Standard search in multiple databases; 1980–2012	Australia	Callahan 2012, Liu 2010, De Silva 2013
Berger, 2014 [45]	Applying evidence mapping techniques to describe the quantity, design, and characteristics of the research on the broad nutrition topic of sugars and health to highlight both what is known and where the gaps exist.	Studies (*n* = 213)	Standard search in Medline; 1946–May 2013	USA	none
Bonell, 2013 [46]	The purpose of the map of evidence and theory and stakeholder consultations was to identify references that are potentially relevant to our review questions, to assess the nature of the references, and to refine our review questions for stage 2. The research questions for this initial mapping stage focused on all aspects of schools’ social and physical environment.	References (*n* = 1,144)	Standard searches in multiple databases; through September 2010	UK	none
Brennan, 2014 [43]	To evaluate the growing literature on policy and environmental strategies to prevent childhood obesity.	Studies (*n* = 600)	Standard search in multiple databases; 2000–2009	USA	Hetrick 2010
Chung, 2011 [39]	The objectives of this technical brief are to describe the current state of use of stress-loading MRI technologies, to enumerate their potential benefits and harms for the diagnosis and management of patients with musculoskeletal disorders for whom this diagnostic test may be considered, and to describe the evidence available to date that supports these applications.	Studies (*n* = 57)	Standard search in MEDLINE; 1975–2010	USA	none
Coast, 2012 [47]	This study systematically maps, assesses, and aggregates research relating to postnatal depression and poverty in low and lower middle income countries.	Studies (*n* = 47)	Standard search in multiple databases; through August 2010	UK	none
Coeytaux, 2014 [48, 49]	To evaluate the existing evidence on yoga for common clinical conditions in Veterans	Reviews (*n* = 10)	Standard search in multiple databases; through July 2014	USA	Arksey 2003, Bragge 2011, Ryan 2009
DeFrank, 2014 [42]	“To understand the extent of evidence on psychological harms, we developed an evidence map that quantifies the distribution of evidence on psychological harms for five adult screening services. We also note gaps in the literature and make recommendations for future research… In characterizing the studies, we gathered information about the study designs, types of measures, and types of outcomes assessed. To focus our study’s scope, we did not assess additional quality indicators of the studies or report their results.”	Studies (*N* = 88)	Standard search in multiple databases; 2002–2012	USA	none
El-Behadli, 2015 [50]	The purpose of this article is to present a map of the extent and distribution of scientific evidence regarding translations of the 9 American Academy of Pediatrics-recommended developmental screening instruments into languages other than English.	Studies (*n* = 64)	Standard search in multiple databases; through June 2014	USA	Arksey 2005, Bragge 2011, Levac 2010
Greer, 2012 [38]	The purpose: “to describe the wheeled mobility service delivery process for patients with complex rehabilitation needs, survey the available literature on service delivery, and identify issues and areas for future research.”	Studies (*n* = 24)	Standard search in multiple databases; through 2011	USA	none
Hempel, 2014 [51]	An evidence map that provides a visual overview of the distribution of evidence (both what is known and where there is little or no evidence base) for mindfulness and a set of executive summaries that would help stakeholders interpret the state of the evidence to inform policy and clinical decision making.	Systematic reviews (*n* = 81)	Standard search in multiple databases and input from experts; through February 2014	USA	none
Hempel, 2014 [41, 58]	The project deliverables are an evidence map that provides a visual overview of the distribution of evidence (both what is known and where there is little or no evidence base) for acupuncture and a set of executive summaries that would help stakeholders interpret the state of evidence to inform policy and clinical decision making.	Publications (*n* = 183)	Standard search in multiple databases; 2005–2013	USA	none
Hempel, 2014 [52]	An evidence map that provides a visual overview of the distribution of evidence (both what is known and where there is little or no evidence base) for Tai Chi and a set of executive summaries that would help stakeholders interpret the state of the evidence to inform policy and clinical decision-making.	Systematic reviews (*n* = 107)	Standard search in multiple databases and input from experts; through February 2014	USA	none
Hitch, 2012 [53]	The purpose of this scoping and mapping project is to assess evidence for the use of focused psychological strategies to enable people with mental health problems to participate in meaningful occupations. In particular, it aims to summarize and thus increases accessibility to the evidence which practitioners could use to support their use of focused psychological strategies to maximize the functional performance and quality of life enjoyed by their clients.	Studies (*n* = 81)	Standard search in multiple databases; since 2000	Australia	Arksey 2005, Bates 2007, Katz 2003
Jaramillo, 2013 [37]	To identify high-priority research questions for osteoarthritis systematic reviews with consideration of health equity and the social determinants of health.	Systematic reviews (*n* = 34)	Preliminary search for a framework on multiple databases (no dates reported); multiple databases for systematic reviews (no dates reported)	Canada	Bragge 2011
Kadiyala, 2014 [54]	This paper comprehensively maps existing evidence along agriculture-nutrition pathways in India and assesses both the quality and coverage of the existing literature.	Articles (*n* = 78)	Standard search in multiple databases, gray literature search; through June 2013	UK	none
Nihashi, 2013 [36]	We constructed an evidence map of clinical evidence on the use of PET in glioma and identified research gaps.	Studies (*n* = 129)	Standard search in multiple databases; through 2011	Japan	Arksey 2005, Hetrick 2010
Northway, 2005 [55]	To collate the evidence for good practice within the context of a wider project into the abuse of people with learning disabilities…it has highlighted common themes, examples of good practice, and the extent and nature of research that can inform adult protection.	*Not reported*	Standard search in multiple databases; no date restricted	UK	none
Sawicki, 2015 [56]	To use evidence mapping to summarize published data on dietary fibers and the human gut microbiome.	Publications (*n* = 153)	Standard search in multiple databases; no dates reported	USA	none
Singh, 2012 [57]	The purpose of this study was to develop a broad synopsis of the available literature through an evidence map of systematic reviews about interventions in adults with prediabetes.	Systematic reviews (*n* = 14)	Standard search in multiple databases and gray literature; January 2012	Canada	Arksey 2005, Hetrick 2010
Vallarino, 2015 [59]	Identify the extent, distribution, and methodological quality of evidence (on the use of psychological interventions for early-stage bipolar disorder in patients aged 15–25 years).	Studies (*n* = 29)	Search in multiple databases and additional sources; no dates reported	UK	Hetrick 2010
Wang, 2015 [60]	To describe the quantity, design, and characteristics of the published studies of low calorie sweeteners and selected health outcomes using evidence mapping.	Studies (*n* = 222)	Standard search on Medline; through July 2014	USA	none
Explicit, online (*n* = 8)					
GEM [16, 62, 64, 65]	To create evidence maps providing an overview of existing research in traumatic brain injury (TBI) and spinal cord injury (SCI)	Publications (*n* = 1644)	Standard search in multiple databases; no time restriction	Australia	Bragge 2011
Headspace [17, 61, 63, 66]	In order to comprehensively and systematically assemble and appraise evidence for a range of treatments across numerous mental health disorders (for young people aged 12–25 years), a mapping methodology was selected.	See below for description from individual publications	See below for description from individual publications	See below for description from individual publications	Arksey 2005, Bragge 2011, Hetrick 2010, Katz 2003, Curran 2007
Callahan, 2012 [63] * headspace*	This paper presents the results of an evidence map we conducted on depression in young people. The extent, range, and nature of high-quality clinical research interventions for depression in young people are summarized.	Publications (*n* = 204, 162 trials, 41 SR/MA)	Standard search in multiple databases; 1980–2009	Australia	Aggregated above
De Silva, 2013 [61] * headspace*	To investigate the extent and nature of research on interventions to prevent and treat suicide and self-harm in young people using evidence mapping.	Reviews (*n* = 6), Studies (*n* = 38)	Standard search in multiple databases; 1980–2011	Australia	Aggregated above
Liu, 2010 [66] * headspace*	Presents an overview of the extent, range and nature of high-quality clinical research interventions for early psychosis by summarizing the empirical evidence from RCTs, CCTs, and SRs and/or MAs.	Publications (*n* = 66, 58 controlled trials, 8 systematic reviews)	Standard search in multiple databases; no dates reported	Australia	Aggregated above
Methodology (*n* = 5)					
Chapman, 2013 [31]	To identify gaps and priorities in the maternal health research, generate a list of maternal health research questions… and adapt questions… to create research questions for use in a future prioritization exercise with experts and other stakeholders.	Systematic reviews (*n* = 178)	Searched the Cochrane Database of Systematic Reviews; 2006–2011	USA	Li et al. 2012, Nasser et al. 2007, de Vet et al. 2001, Clarke et al. 2007
Curran, 2007 [33]	What empirical evidence is available on the relationships between mental health problems and social exclusion? What is the nature of this evidence? Is it qualitative or quantitative? Which mental health and social exclusion topics are well researched and which are not? Which countries are the studies set in? What research designs are used to generate the evidence?	Publications (*n* = 72 includes from a random sample of 200 pulled from 16,115 total search hits)	Search in multiple databases; 1948–2003	USA	Gough and Elbourne 2002
Frampton, 2014 [34]	To assess the effectiveness and cost-effectiveness of educational interventions for preventing catheter-blood stream infections in critical care units in England.	Studies (*n* = 74)	Standard search in multiple databases; through 2011	UK	Shepherd et al. 2010, Shepherd et al. 2006, Rees et al. 2006
Katz, 2003 [18]	To map the evidence pertaining to many commonly used complimentary and alternative medicine practices.	Publications (only presented counts by category, mutual exclusivity not addressed, all study designs and included systematic reviews/ meta-analyses)	Standard search in multiple databases, gray literature included; through 2000/2001	USA	None
Wysocki, 2007 [32]	To provide an overview of the key issues and evidence map related to the use of whole-body vibration therapy for the prevention and treatment of osteoporosis.	Studies (*n* = 12)	Standard search in multiple databases and gray literature; through 2010	USA	None

^a^For publications from more than one country, the country of the corresponding author was used

Although a variety of topics are represented in the 39 included evidence map publications, four broad topical areas account for nearly three quarters of the publications: mental health and learning disability related topics account for 11 of the publications (28 %), complimentary alternative medicine and nutrition-related topics account for seven publications each (18 % each), and traumatic brain injury and spinal cord injury account for four publications (10 %). Within the mental health group, four of the 11 publications come from the headspace research group, described in more detail below. Six of the seven complementary and alternative medicine publications come from the Veterans Affairs Evidence Synthesis Program, of which the authors of this review are a part. Three of the seven nutrition-related publications come from a research group based at Tufts University, and all four of the traumatic brain injury/spinal cord injury publications are a part of the Global Evidence Mapping (GEM) project. Taken together, these four research groups account for 44 % of the published evidence maps included in this review (17/39).

### Definition of evidence map

Of the 39 included studies, eight were associated with two evidence mapping projects: GEM [16, 62, 64, 65] and headspace [17, 61, 63, 66]; these publications were grouped by project in the following discussion since publications from the same project employ the same definition. In two instances, there were two publications from a single mapping project, and these were also grouped, resulting in two evidence maps from these four publications [41, 48, 49, 58]. One publication provided neither definition nor citation [32]. Most studies did not explicitly outline a definition of “evidence map,” and often the research aims were used to capture an implicit definition (see Table 1 for research questions or aims for all included studies).

Thus, of the 31 evidence maps with elements of a definition, the most commonly stated component of the definition was a review of evidence to identify gaps or future research needs (67 %, 21/31). Another common component was that the process engage the audience and/or produce user-friendly products (58 %, 18/31). In emphasizing the user-friendly aspect, one definition stated that evidence maps should “provide access to user-friendly summaries of the included studies” [15], while another described evidence maps as “mak[ing] these vast bodies of literature accessible, digestible, and useable” [44]. Many definitions also qualified evidence maps as capturing a broad field (55 %, 17/31). Two components were less often explicitly stated in the definition, having a systematic process and visual depiction (48 %, 15/31 and 23 %, 7/31, respectively), but all included publications used a systematic process (i.e., documented search strategy and inclusion criteria) and most incorporated a visual depiction of the data as well (84 %, 26/31).

Only one evidence map definition explicitly stated all five of these components (i.e., identify gaps or needs, audience engagement/user-friendly products, broad field, systematic process, and visual depiction) [49]. Three other evidence maps met all criteria when their inclusion of a visual depiction was considered a part of the definition implicitly [39, 57, 63]. Seventeen of the evidence maps included four of the five definition components, including those that used a systematic process or visual depiction without explicitly stating this [16, 35–38, 40–42, 44–47, 51–53, 56]. Thus, 68 % (21/31) evidence maps with definitions included four or five of the five most common components.

Most of the 31 evidence maps did not provide any citations when referencing evidence mapping methodology (*N* = 15). Citations occurring more than twice included Arksey and colleagues [67] (*N* = 8), the GEM project [16] (*N* = 7), the headspace project [17] (*N* = 5), and Katz and colleagues [18] (*N* = 3). The publication by Arksey and colleagues provides a description of scoping review methods but was still the most cited article. The GEM and headspace projects are both large initiatives that have funded the exploration of a very broad topic, neurotrauma and mental health disorders affecting youth, respectively. The publication by Katz and colleagues, the oldest of the publications we identified, was published in 2003, at least 4 years before any of the other identified mapping publications [18]. This publication introduces the term, as well as a nine-step process: identify and convene the appropriate experts, apply expert opinion to define the region of evidence to be mapped, establish the coordinates to be used for positioning within the map, define the map boundaries in terms of pertinent coordinates, search the relevant “terrain,” draw the map, study the map to identify any needed revisions and to establish priorities for detailed assessments, perform detailed assessments in priority areas, and generate reports summarizing the “lay of the land.”

### Domains used to classify data in evidence maps

Only publications that presented an evidence map as a figure or online database were considered relevant in determining domains, or data elements used to classify literature, since these publications were the ones with data presented for such domains. Of the 34 publications, 26 presented the maps in the publication itself, and eight were associated with an online map. As in the definition section, duplicate publications from the same evidence mapping project were collapsed together, leaving a total of 26 evidence maps with domains for this synthesis.

Thirteen evidence maps categorized their literature by the amount of literature relevant to a particular domain (literature size domain, 50 %, see Table 2). Other popular domains included intervention (*N* = 12, 46 %), study design (*N* = 10, 38 %), sample size (*N* = 10, 38 %), disorder/condition (*N* = 9, 35 %), and outcomes (*N* = 9, 35 %). Some maps grouped literature in subdomains within these larger domains, such as groups of conditions and then individual conditions within those groups. The average number of domains captured in an evidence map was four, the smallest number being two (i.e., outcomes and elements of service delivery) and the largest being seven (i.e., population characteristics, intervention, outcomes, setting, study design, sample size, and disorder/condition).

**Table 2:** Evidence map presentations and domains used to classify data in the evidence maps

Author, year	Evidence map presentations	Classic PICOTS						Study design	Sample size (N)	Disorder/condition	Systematic review domains			Other, specify
		Population characteristics	Intervention	Comparators	Outcomes	Timing	Setting				Literature size	Estimated effect/association	Confidence in estimate	
	Title: description of map(s); all identified studies represented													
Explicit, published (*n* = 26)														
Althuis, 2013 [35]	“Evidence map of publications of sugar-sweetened beverages by outcome and study type”(F2): flow diagram; yes, “Evidence map of published cohort and intervention studies of sugar-sweetened beverages by outcome and key study features” (F3): flow diagram and cross-tabular table hybrid; yes	x			x	x	x	x	x					
Antsee, 2011 [40]	“The matrix” (F1): cross-tabular table with color-coded subdivisions in each cell; yes	x						x						Prevention area: several important areas within HIV prevention research that represent potential for novel and innovative research of interest (e.g., education, behavior, service delivery, descriptive epidemiology, international adaptability, etc.)
Bailey, 2014 [44]	“Distribution of included prevention studies” (T1), “Distribution of included disorder established treatment studies” (T2), “Distribution of included relapse prevention studies” (T3): cross-tabular table; each table is a subset		x					x		x				Intervention type: larger categories interventions fall within (e.g. psychological, biological, service, universal, at-risk)
Berger, 2014 [45]	"Frequency of intervention comparisons within outcome groups, by baseline health status in trials" (F2), "Frequency of intervention comparisons among cardiometabolic outcomes, by baseline health in trials" (F3): bubble plot using color and bubble size; yes	x		x	x						x			
Bonell, 2013 [46]	"Countries of primary research of studies included in the evidence map"(F3), "Health topics of the references included in the evidence map" (F4), "School/grade level of the references included in the evidence map" (F5), "Aspect of the school examined in the references included in the evidence map" (F6): bar chart, yes						x				x			Health topic
Brennan, 2014 [43]	“Example evidence map for associational studies for childcare food and beverage policies and environments” (F2), “Example evidence map for intervention studies for childcare food and beverage policies and environments” (F3): conceptual model mapping strategy to outcomes; no, example only		x		x							x		Short/intermediate/long-term outcome groups
Chung, 2011 [39]	“Studies stratified by design and anatomic region imaged” (F4), “Studies stratified by design and device category” (F5): bubble plot and cross-tabular hybrid, within each cell bubbles of varying size and color; yes		x					x	x					
Coast, 2012 [47]	"Relationships between postnatal depression and poverty identified in the mapping" (T3): cross-tabular table; yes						x	x	x		x	x		Poverty indicator
	Also present table of study characteristics (T1); yes													
Coeytaux, 2014 [48, 49]	"Characteristics of Systematic Reviews Evaluating Yoga for All Eligible Conditions" (T1): cross-tabular table; yes							x	x	x	x			SR quality, SR methods
	"RCTs evaluating yoga" (F2): bubble plot with bubble size; yes													
DeFrank, 2014 [42]	“Number of studies assessing categories of psychological harms and rates of overdiagnosis” (F2): bar graph with color-coded subdivisions; yes		x								x			Assessing burden/frequency/both
El-Behadli, 2015 [50]	"Evidence in Peer-Reviewed Publications of Translation Methods" (T2), "Evidence in Peer-Reviewed JournalsRegardingRestandardization of Translations" (T3): cross-tabular table; yes													Language, screener translated, translation methods
Greer, 2012 [38]	“Summary of studies on wheeled mobility service delivery” (T2): Cross-tabular table; yes				x									Elements of service delivery: factors important to individuals when considering wheeled mobility options, children’s caregivers’ and parents’ opinions about the wheeled mobility used by their child, user satisfaction
Hempel, 2014 [51]	"Evidence map of mindfulness": bubble plot; yes		x						x	x	x	x		
Hempel, 2014 [41, 58]	“Evidence map of acupuncture for pain” (F3), “Evidence map of acupuncture for wellness” (F4),“Evidence map of acupuncture formental health” (F5): bubble plots with color and bubble size as dimensions in addition to x and y axes; each diagram is a subset									x	x	x	x	
Hempel, 2014 [52]	"Evidence map of tai chi" (F2): bubble plot; yes								x	x	x	x		
Hitch, 2012 [53]	"Available evidence by diagnosis and focused psychological therapy" (T2), "Availableevidence by diagnosis and level of evidence" (T3), "Quality of evidence by intervention" (T4): cross-tabular format; yes		x							x			x	
Jaramillo, 2013 [37]	“Map of Evidence for Osteoarthritis Template” (F3): cross-tabular table; no Online appendix version has research questions all mapped to grid													Studies are not classified this map classifies research question developed from workshop discussions
Kadiyala, 2014 [54]	"Mapping the agriculture-nutrition pathways in India"(F1) with "Number of studies included in the evidence review by agriculture-nutrition pathways and study design" (T2): conceptual model with companion cross-tabular display; yes				x		x	x			x			pathways between factors
Nihashi, 2013 [36]	“Current clinical evidence on PET in glioma” (F2): three dimensional cross-tabular visualizationusing color and stacked discs of varying size; yes		x	x					x					
Northway, 2005 [55]	"Examples of key concerns and good practice" (T2): cross-tabular; examples only													
Sawicki, 2015 [56]	"Microbiome Outcomes Examined by Fiber Type" (F4), "Other Health Outcomes Examined with the Microbiome by Fiber Type" (F5): bubble plots, yes		x		x				x		x			
Singh, 2012 [57]	"Interventions for prediabetes investigated in systematic reviews" (T4), "Outcomes assessed in systematic reviews of prediabetes" (T5), "Ratings of authors’ overall conclusions about interventions"(T6): cross-tabular, yes		x		x						x	x	x	
Vallarino, 2015 [59]	"Evidence map of all 29 studies of psychological interventions for the early stages of bipolar disorder" (F2): flow chart; yes		x							x	x			
Wang, 2015 [60]	"Bubble Plot of LCS Studies by Study Duration and by Health Outcome Groups" (F3): bubble plot, yes	x			x			x	x		x			Study duration
	"Study Design and Population Characteristics" (T2): cross-tabular, yes													
Explicit, online (*n* = 8)														
GEM [16, 62, 64, 65]	“Example of ‘interventions and study design output’” (T3): Cross-tabular table; no, “example only”, “Example of ‘detailed study characteristics output (extract only)’” (T4): evidence table; no “example only	x	x		x		x	x	x	x				
Headspace [17, 61, 63, 66]			x					x		x				
Callahan, 2012 [63]	“Distribution of included universal preventive studies” (F2), “Distribution of included indicated and selective preventive studies” (F3), “Distribution of included studies to treat a diagnosed depressive disorder” (F4): Flow diagram; each diagram is a subset													
De Silva, 2013 [61]	“The distribution of included trials in categories during second-stage screening” (F2): flow diagram; yes													
Liu, 2010 [66]	“Distribution of included… studies” (F2): flow diagram; yes													

### Form the evidence map takes once completed

Three main versions of an evidence map were found: publications with a visual representation of data in the publication (*N* = 26); publications that referenced a database housing data virtually that can be queried (*N* = 8); and publications that employed a process or methodology leading to recommendations or synthesized result (*N* = 5).

### Visual representation of data

Twenty-six publications explicitly include figure(s) or table(s) which are referred to as evidence maps. They display a range of potential map formats, some quite similar to a classic systematic review evidence table or literature flow diagram. These formats incorporate various numbers of characteristics and types of characteristics. Details of map formats can be found in Table 2. As in prior sections, two projects with two publications each are collapsed into two groups [41, 48, 49, 58], and thus, 24 evidence maps are discussed in this section.

**Fig. 3:**
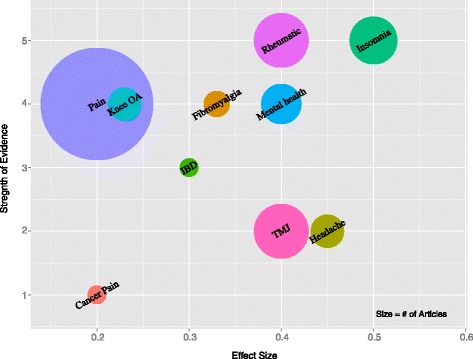
Evidence map of acupuncture for pain. The *bubble plot* shows an estimate of the evidence base for pain-related indications judging from systematic reviews and recent large trials. The plot depicts the estimated number of RCTs (size of the bubble), the effect size (*x*-axis), and the strength of evidence (*y*-axis)

Most of the 24 published evidence maps used some variant of a cross-tabular format for their main findings, with counts or sums of publications arrayed across various domains (*N* = 10, 42 %). Eight bubble plots (33 %), two flow charts (8 %), and two bar charts (8 %) were used as the main findings diagram in evidence mapping publications. The last two publications arrayed included studies on a conceptual framework (8 %) and mapped the evidence on the relationships described in the model. Most publications presented data in more than one table or graphic (*N* = 16, 67 %), either with subsets of data in each map (e.g., one table for condition A and another for condition B) or with different domains covered in the different maps (e.g., one graphic describing population demographics covered by the literature and another with outcomes cross-tabulated with interventions of included studies). Figure 3 presents a figure simulating a bubble plot style evidence map as an example of one of the more commonly used formats.

### Map as online database

Eight publications discuss evidence maps in the context of online searchable databases. Four publications discuss GEM [16, 62, 64, 65] and four publications discuss headspace [17, 61, 63, 66]. Of the four publications discussing GEM, one provides the overview of the initiative and lays out the GEM mapping method [16], one discusses search strategies that the GEM group has developed [65], and the final one discusses the process of developing priority research areas for which evidence maps can be generated [62]. For this group of publications, there is one product: the searchable online GEM database.

In the case of the headspace publications, there is an overview publication that lays out the history and scope of the headspace program and their mapping methods [17], but each of the three additional publications presents data on a subsection of the overall evidence map [61, 63, 66]. While the overall program aims to improve evidence-based practice in youth mental health, the subtopics of interventions for depression, suicidal and self-harming behaviors, and psychosis are explored in depth in the latter publications, making it less clear if the overall database is the evidence map, or if the data presented within the topic-specific publications are the evidence maps. Although none of the figures are titled “evidence map,” they appear similar to some of those included in the prior section.

### Use of evidence mapping as methodology

Five publications used a mapping methodology without presenting an explicit map [18, 31–34]. With or without producing either of the first two conceptions of an evidence map, the third idea was presented or discussed in all included publications. The process, either discussed or executed, usually included a search of multiple databases with standard search terms, discussion of inclusion criteria, and inclusion of stakeholders in defining and/or refining the scope of the product.

## Discussion

The principal conclusion of our evaluation of studies that call themselves “evidence maps” is that the implied definition, as defined by a majority of studies reporting individual components, of what constitutes an evidence map is a systematic search of a broad field to identify gaps in knowledge and/or future research needs that presents results in a user-friendly format, often a visual figure or graph, or a searchable database. However, only four of 31 studies (12 %) satisfied all five of these components. Thus, the heterogeneity in methods and stated definitions means that stakeholders cannot necessarily know what to expect if they commission an evidence map or seek to identify existing maps.

Of all literature synthesis methods, this evidence mapping definition shares many similarities with the definition or goals of a scoping review. Both seek to review broad topics, often with the stated goal of identifying research gaps and areas for future research. In evidence mapping publications, the most often cited article for evidence mapping methods is a scoping methods publication [67]. Compared to a scoping review, using the framework suggested by Colquhoun and colleagues [3], the methods used in the evidence mapping publications are, on a whole, very similar. The main distinctions seem to be the involvement of stakeholders early in the research process, the rigor of the search strategy (e.g., all mapping publications describing systematic searches of online databases), and the production of a visual or searchable database, with the stated goal that such products are more “user-friendly” or digestible. Because neither methodology has established reporting guidelines, it is difficult to determine where one method ends and the other begins.

In terms of the “map,” the most common ways of organizing the data into a visual representation were using a cross-tabular format and categorizing literature according to interventions and/or study designs present. However, the domains chosen to display and means of presentation will necessarily vary for any particular map according to the aims of the review.

### Limitations

Because there is no standard search term or repository for evidence maps, we may not have been able to identify all evidence maps through our search methods. Thus, our findings may be biased to represent those maps that were readily available on the ten searched databases or were cited in other sources we found.

### Future research

With growing numbers of publications using the term “evidence map,” clarifying scoping and evidence mapping methods is an important topic for stakeholders so that they will know what to expect when commissioning, conducting, and interpreting results of an “evidence map.” A key part of this effort will be developing reporting guidelines for these methods. This is already underway for scoping reviews [3], but given the similarities between the two methods, working on both methods in tandem may produce more clear and distinct results.

A key strength of the evidence mapping method is the use of visuals or interactive, online databases. Keeping these data up to date and available online may prove to be a challenge in rapidly growing fields, and new audiences may be exposed to these resources who are unfamiliar with how to best make use of the products. As evidence synthesis methods evolve to meet modern demands, they must also meet new challenges.

## Conclusions

The principal conclusion of our evaluation is that the implied definition of what constitutes an evidence map is a systematic search of a broad field to identify gaps in knowledge and/or future research needs that presents results in a user-friendly format, often a visual figure or graph, or a searchable database. However, there is diversity in methods and stated definitions, so that, as it stands now, stakeholders cannot necessarily know what to expect if they commission an evidence map or seek to identify existing maps.

## Additional files

Additional file 1:
**PRISMA Checklist.** (DOC 63 kb) Additional file 2:
**PRISMA Flow. (**DOC 57 kb)

## Abbreviations

GEM: Global Evidence Mapping.
MeSH: Medical Subject Headings
PROSPERO: International Prospective Register of Systematic Reviews
