# Supplementary material for: What is an evidence map? A systematic review of published evidence maps and their definitions, methods, and products
Source: Syst Rev. 2016 Feb 10;5:28. doi: 10.1186/s13643-016-0204-x (PMC4750281; doi:10.1186/s13643-016-0204-x)
Supplement: Additional file 2: — PRISMA Flow. (DOC 57 kb) [file 13643_2016_204_MOESM2_ESM.doc]

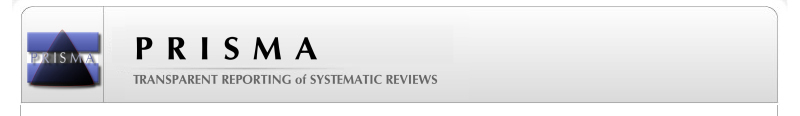
**PRISMA 2009 Flow Diagram**

**Screening**

**Included**

**Eligibility**

**Identification**

Records identified through database searching
(n = 145 )

Additional records identified through other sources
(n = 2 )

Records after duplicates removed
(n = 147 )

Records screened
(n = 53 )

Records excluded
(n = 94 )

Full-text articles assessed for eligibility
(n = 53 )

Full-text articles excluded, with reasons
(n = 14 )

4: other synthesis method identified published title

3: Useful for background discussion but do not present evidence map

2: use of term “evidence map” but not evidence synthesis

2: used data from separate evidence map project

1: used systematic review protocol/guidelines

1: Duplicated citation

1: Full text not available

Studies included in qualitative synthesis
(n = 39 )

Studies included in quantitative synthesis (meta-analysis)
(n = 0 )
